# Relation between Red Cell Distribution Width and Left Ventricular Function in Children with Heart Failure

**DOI:** 10.1155/2014/234835

**Published:** 2014-02-10

**Authors:** Wegdan Mawlana, Amr Donia, Doaa Elamrousy

**Affiliations:** Department of the Pediatrics, Tanta University Hospital, Tanta 31125, Egypt

## Abstract

*Background*. Most of the studies done on adults showed that red cell distribution width (RDW) can be used as a prognostic marker in patients with chronic heart failure. However, RDW has not been tested in children with heart failure. *Methods and Results*. 31 children with heart failure admitted to Cardiology Unit, Tanta University Hospital, during the period of January 2012 to December 2012 were included in this study, RDW as a component of routine blood count was evaluated and correlated to the echocardiographic parameters of left ventricle. The mean age of our cohort was 16.16 ± 14.97 months, congenital heart disease with left-to-right shunt represented 58.1% of the underlying causes of heart failure while dilated cardiomyopathy made 41.9%. The mean hemoglobin level was 9.14 ± 1.18 gm/dL; RDW level ranged from 10.7% to 27.7% with a mean of 16.01 ± 3.34. Hemoglobin was significantly correlated with RDW at any level. For the echo parameters, at cutoff point of 16.4%, RDW was significantly correlated with fraction shortening (FS), and *A, E/A* ratio, but it was not correlated with LVEDD, LVESD, and *E/É* at the same cutoff level. *Conclusion*. RDW, a simple, available test, can be used as a marker for the left ventricular function in children with heart failure until an echocardiography assessment for the patients is done.

## 1. Background

Red cell distribution width (RDW) measures the variation in the size of erythrocytes. It has been used to differentiate iron deficiency anemia from thalassemia trait, where increased RDW is associated with iron deficiency anemia [1, 2]. The association between anemia and cardiovascular outcome has been studied in the adult population. RDW has been recently discovered as a new marker in heart failure. It was found that increased RDW was associated with increased morbidity and mortality in chronic heart failure [3]. There is no definite pathophysiology explaining this association. Several factors may be proposed including inflammation, nutritional deficiencies, and inadequate production of erythropoietin [4–6]. Congestive heart failure is the most common cause for admission to the Pediatric Cardiology Unit at Tanta University Hospital. Congenital heart diseases with left-to-right shunt as VSD and PDA represent the majority of cases with heart failure; acute exacerbation of chronic heart failure in children with dilated cardiomyopathy forms another group. Several cardiac biomarkers have been tested as predictors for the severity of heart failure including brain natriuretic peptide, N-terminal pro-BNP (NT-proBNP), and cardiac troponins [7, 8]. RDW is a component of routine complete blood count done for all patients admitted with the diagnosis of heart failure. However, the relation between RDW and left ventricular function assessed by echocardiography has not been studied in children.

## 2. Patients and Methods

This is a prospective randomized study done on 31 children who were admitted to the Pediatric Cardiology Unit at Tanta University Hospital during the period from January 2012 to December 2012 with clinical diagnosis of heart failure. Study approval was obtained by the local ethics committee. For all patients, Complete blood count with RDW was measured with the use of an analyzer on the day of admission.

Two-dimensional echocardiography was performed and analyzed in all the patients on admission using Ultrasound Machine, Vivid 7 (GE) medical system, Horten, Norway with 3.5 multi-frequency transducer. Fractional shortening was measured using the equation (FS = Dd − Ds/Dd × 100) where Dd is the end-diastolic dimension of the left ventricle (LV) and Ds is the end-systolic dimension of the LV.

Pulsed-wave Doppler echocardiography of the mitral inflow was performed for all the children to measure peak velocity of early diastolic filling (*E*), late filling (*A*). All the children underwent tissue Doppler imaging (TDI) to measure early diastolic mitral annular velocity (*É*) and (*E*/*É*) ratio was calculated.

## 3. Statistical Analysis

The relation between different values of RDW and various echocardiographic parameters were tabulated and statistically analyzed to explore any significant results. Numerical variables were presented using mean and standard deviations; categorical variables were described using numbers and percentages. Comparisons of continuous variables were done using the unpaired Students *t*-test. Correlations of RDW with various echo parameters were examined by Pearson correlation analysis. For RDW we used 16.4% (median) as a cutoff point to examine if RDW above 16.4% was useful to predict impaired left ventricular function. *P* < 0.05 was considered statistically significant.

## 4. Results

Tables 1 and 2 demonstrate the baseline characteristics of our cohort with heart failure. Our study group consisted of 31 children with male sex representing 38.7% (12 males). Their age ranged from 2 months to 60 months with mean of 16.16 ± 14.97 months. 41.9% of our patients were diagnosed as dilated cardiomyopathy while congenital heart disease (CHD) with left-to-right shunt representing 58.1%. Isolated VSD formed the main CHD among the studied group (26.81%). Hg ranged from 7.2 gm/dL to 12.2 gm/dL with mean of 9.14 ± 1.18 gm/dL. The mean value of RDW was 16.01 ± 3.34 with the range of 10.70 to 27.7%. There were no significant differences between male and female children with respect to the age and different laboratory and echocardiographic parameters (Table 3).

This study investigated the relation between RDW and different laboratory and echo parameters as presented in Table 4. The optimal cutoff value of RDW was 16.4% (median); RDW level was significantly related to hemoglobin level (*P* = 0.047), fraction shortening (FS) (*P* = 0.003), *A* (*P* = 0.12), and *E/A* ratio (*P* = 0.012). However, age (*P* = 0.058), LVEDD (*P* = 0.170), LVESD (*P* = 0.156), and *E* (*P* = 0.123) were not significantly related to RDW level.

We studied if RDW level ≥16.4 was associated with higher *E*/*É* in our group; however there was no significant correlation between *E*/*É* and RDW at a level of ≥16.4%.  Table 5 summarizes the echo parameters that significantly correlated with RDW.

## 5. Discussion

A wide variety of biological markers have been used as a predictive for morbidity and mortality in patients with heart failure; however, many of them are still used for research only [9].

Evaluation of routine laboratory values as marker for heart failure has not been given much interest. red cell distribution width (RDW), a variation in the size of erythrocytes, has been investigated as a new marker in cardiac disease [10]. It is readily available with routine CBC. RDW is elevated either due to impaired production or increased destruction of erythrocytes.

The interaction between heart failure and hematological system is not well understood. Among the several potential causes of this association, inflammatory cytokines as TNF-**α** as well as IL-6 have been shown to be predictors in heart failure; these cytokines may impact bone marrow function and iron metabolism. This supports the basic role of inflammation in the induction of ineffective red cell production, reflected by increased RDW values [11].

Most of the studies investigated RDW as prognostic marker in adults with chronic heart failure [12, 13]. To our knowledge, none of these studies focused on heart failure within the pediatric age group. Our cohort included children admitted with heart failure. congenital heart diseases with left-to-right shunt representing 58.1%, with VSD represent 26.7%, then comes dilated cardiomyopathy as the second cause of heart failure in the studied group (41.9%).

Our study showed that RDW correlated significantly with hemoglobin, this in in agreement with the study done by Felker et al. 2007, which showed that RDW and hemoglobin were negatively correlated with each other. Previous studies examining the relationship between hemoglobin and severity of cardiac failure showed that low hemoglobin was associated with adverse outcome. Most recently Increased RDW was shown to be a strong predictor of increased morbidity and mortality in patients with chronic heart failure [3]. In view of these studies, we can use RDW as a prognostic factor for our patients but we should be cautious when interpreting these results as most of these studies were conducted in adults with chronic heart failure.

Other principal findings in this study are that the RDW level at cutoff point of 16.4% was significantly correlated with the echo parameters such as FS, and *A* velocity, *E*/*A* ratio, and was not correlated with the *E* velocity and *E*/*É* ratio. These results are in concordance at some points with the study done by OH et al., 2009 [14], which reported significant correlation between RDW and echo parameters such as E velocity, and *E*/*É* ratio. Our study did not show correlation with *E*/*É* ratio which could be explained by the small number of our cohort.

The current study has some limitations that should be considered when evaluating our results. Small number of the studied group may impact the results; large numbers of patients would strengthen the results, further study needed to correlate serial RDW with the clinical severity of heart failure as well as follow-up of the patient as regards frequent hospitalization and or death.

In conclusion, RDW, which is routinely measured and reported as a component of the standard complete blood count, was significantly correlated with hemoglobin and echo parameters for evaluation of left ventricular function in children with heart failure. This emphasizes that elevated RDW can be used as a novel marker in children with heart failure. This study should prompt further evaluation of the association between RWD and outcome in children with heart failure to improve understanding of the pathophysiology of heart failure. We suggested the RDW as a simple test can be used as a marker for heart failure in emergency room.

## Figures and Tables

**Table 1:** Demographic characteristics and etiology of heart failure in study group.

	*N*	%
Sex		
Male	12	38.71
Female	19	61.29
Cause of Heart Failure		
VSD/ASD	5	16.13
DCM	13	41.94
ASD	1	3.23
VSD	8	26.81
PDA/VSD	2	6.45
PDA	1	3.23
CAVC	1	3.23

VSD: ventricular septal defect; ASD: atrial septal defect; DCM: dilated cardiomyopathy; DA: patent ductus arteriosus; CAVC: common atrioventricular canal.

**Table 2:** Laboratory and echocardiographic parameters of children with heart failure.

	Range	Mean ± SD
Age (Month)	2.000–60.000	16.161 ± 14.971
Hb (gm)	7.200–12.200	9.142 ± 1.180
RDW (%)	10.700–27.700	16.019 ± 3.348
FS (%)	7.220–51.700	24.974 ± 14.512
LVEDD (cm)	1.600–5.100	3.206 ± 1.403
LVESD (cm)	0.900–4.600	2.516 ± 1.523
*E* (m/s)	0.390–1.000	0.699 ± 0.181
*A* (m/s)	0.280–1.290	0.624 ± 0.342
*E*/*A*	0.600–2.000	1.328 ± 0.514
$E/\overset{´}{E}$	0.750–17.500	12.339 ± 4.538

Hb: hemoglobin; RDW: red cell distribution width; FS: fraction shortening; LVEDD: left ventricular end diastolic dimension; LVESD: left ventricular end systolic dimension; *E*: peak velocity of early diastolic filling; *A*: peak velocity of late filling; $E/\overset{´}{E}$: early mitral inflow velocity to early diastolic mitral annular velocity ratio.

**Table 3:** The difference between both sexes in laboratory and echo-parameters.

	Male	Female	*t*-test	
	Mean ± SD	Mean ± SD	*t*	*P*-value
Age (month)	8.667 ± 7.152	20.895 ± 16.769	−2.381	0.024
Hb (gm/dL)	9.533 ± 1.476	8.895 ± 0.906	1.498	0.145
RDW (%)	15.758 ± 4.772	16.184 ± 2.166	−0.340	0.736
FS (%)	30.889 ± 12.880	21.238 ± 14.544	1.878	0.070
LVEDD (cm/s)	2.350 ± 0.799	3.747 ± 1.446	−3.054	0.005
LVESD (cm/s)	1.675 ± 0.730	3.047 ± 1.665	−2.684	0.012
*E* (cm/s)	0.691 ± 0.229	0.704 ± 0.151	−0.189	0.851
*A* (cm/s)	0.728 ± 0.362	0.558 ± 0.320	1.361	0.184
*E*/*A*	1.080 ± 0.491	1.485 ± 0.476	−2.283	0.030
$E/\overset{´}{E}$	12.117 ± 4.045	12.479 ± 4.926	−0.213	0.833

Hb: hemoglobin; RDW: red cell distribution width; FS: fraction shortening; LVEDD: left ventricular end diastolic dimension; LVESD: left ventricular end systolic dimension; *E*: peak velocity of early diastolic filling; *A*: peak velocity of late filling; $E/\overset{´}{E}$: early mitral inflow velocity to early diastolic mitral annular velocity ratio.

**Table 4:** Laboratory and echocardiographic parameters stratified by RDW value.

	<16.4	>16.4	*t*-test	
	Mean ± SD	Mean ± SD	*t*	*P* value
Age (month)	21.400 ± 18.616	11.250 ± 8.466	1.976	0.058
Hb (gm/dL)	9.573 ± 1.015	8.738 ± 1.209	2.077	0.047*
FS (%)	17.426 ± 9.957	32.050 ± 14.773	−3.209	0.003*
LVEDD (cm/s)	3.567 ± 1.262	2.869 ± 1.482	1.407	0.170
LVESD (cm/s)	2.920 ± 1.428	2.138 ± 1.556	1.456	0.156
*E* (cm/s)	0.647 ± 0.148	0.748 ± 0.200	−1.586	0.123
*A* (cm/s)	0.448 ± 0.125	0.789 ± 0.398	−3.166	0.004*
*E*/*A*	1.562 ± 0.527	1.109 ± 0.404	2.693	0.012*
$E/\overset{´}{E}$	13.800 ± 3.680	10.969 ± 4.940	1.800	0.082

*Significant.

Hb: hemoglobin; RDW: red cell distribution width; FS: fraction shortening; LVEDD: left ventricular end diastolic dimension; LVESD: left ventricular end systolic dimension; *E*: peak velocity of early diastolic filling; *A*: peak velocity of late filling; $E/\overset{´}{E}$: early mitral inflow velocity to early diastolic mitral annular velocity ratio.

**Table 5:** Factors affecting RDW value.

	<16.4	>16.4	*t*-test	
	Mean ± SD	Mean ± SD	*t*	*P* value
Hb (gm/dL)	9.573 ± 1.015	8.738 ± 1.209	2.077	0.047*
FS (%)	17.426 ± 9.957	32.050 ± 14.773	−3.209	0.003*
*A*	0.448 ± 0.125	0.789 ± 0.398	−3.166	0.004*
*E*/*A*	1.562 ± 0.527	1.109 ± 0.404	2.693	0.012*

*Significant.
